# Immediate impacts of the COVID-19 pandemic on household economic activities and food security in Tajikistan

**DOI:** 10.1007/s41885-021-00104-4

**Published:** 2022-01-17

**Authors:** Enerelt Murakami

**Affiliations:** Japan International Cooperation Agency, Ogata Sadako Research Institute for Peace and Development, 10-5, Ichigaya Honmuracho, Shinjuku-ku, Tokyo, 162-8433 Japan

**Keywords:** COVID-19, Employment, Household income, Food security, Event study designs, Coping mechanisms

## Abstract

Using a nationally representative monthly survey, administered both before and after the outbreak of the COVID-19 pandemic, this paper provides estimates of household responses to the COVID-19 pandemic in Tajikistan, focusing on (i) short-term dynamic impacts on household economic outcomes and food security, (ii) heterogenous effects across different households, and (iii) coping with income shocks resulted from the pandemic. Parametric and non-parametric event studies are estimated to quantify the short-run dynamic impacts of the pandemic on household activities. The findings show that household employment and income dropped, and food insecurity immediately worsened with the first confirmed COVID-19 cases and continues to deteriorate six months into the pandemic in Tajikistan. The extent of the impacts varies depending on locations, pre-pandemic income levels, and household sizes. In response to the income shock brought about by the pandemic, households increased borrowings and reduced food and health expenditures. These results are robust to different specifications.

## Introduction

The ongoing corona virus pandemic (COVID-19) caused by severe acute respiratory syndrome coronavirus 2 (SARS-Cov-2) has triggered unprecedented health crises and economic disruption globally. By the end of 2020,^1^ there had been over 81 million confirmed cases with 1.8 million COVID-19-related deaths globally.^2^ The rapid spread of the virus has led countries around the world to adopt various measures to contain and prevent the spread of COVID-19, including lockdowns, social distancing, closures of businesses and schools, and prohibitions of mass gatherings. In addition to the direct health impacts, these containment measures have had a substantial impact on local businesses, employment, income, poverty, health, education, and food security.

While the overall impacts of the pandemic are yet to be determined, this paper aims to provide estimates of the immediate impacts of COVID-19 on household economic activities and households’ responses to the income shock caused by the pandemic in Tajikistan. The household activities that this paper takes into consideration are employment, income, migration, remittances, and food security.^3^ More specifically, this paper investigates the following: (i) short-run impact of the pandemic, (ii) heterogenous effects across location, pre-pandemic income and household size, and (iii) coping mechanisms with the income shock brought by the pandemic.

Due to its large informal sector, high dependency on migrant remittances, and no government mandated lockdowns during the pandemic, Tajikistan provides an interesting case study on the impacts of the ongoing pandemic. As of 2018, more than 42% of the country’s total working-age population were neither studying nor working (United Nations Development Programme 2020). These people are likely to be engaged in the informal economy and do not have job security. Given the lack of job creation in Tajikistan, many people migrate abroad, particularly to Russia, to work (Japan International Cooperation Agency 2018). Remittances sent back home by these migrants fueled the economy and have helped alleviate poverty over the past two decades (World Bank 2020a). At the same time, the dependency on remittances translates into a vulnerability of the Tajik economy to external shocks, especially those from Russia. The pandemic has further exacerbated these job insecurities and vulnerabilities to external shocks. Restrictions on human mobility and economic activities at home and abroad resulted in a sharp drop in remittances and employment (World Bank 2020b). Although Tajikistan has not imposed any lockdowns, prolonged declines in household income due to loss of remittance receipts and income from employment could reverse the success the country has had in alleviating poverty over the last 20 years.

To quantify the immediate impacts of the COVID-19 pandemic on household activities, I apply parametric and non-parametric event studies. The data used in this paper come from an ongoing nationally representative monthly survey, Listening to Tajikistan (L2TJK), that has been implemented both before and after the outbreak of the COVID-19 pandemic. In analyzing the immediate or short-run impact of the pandemic, the dynamic effects are first visualized using the non-parametric event studies. Then, based on the patterns observed in the non-parametric event study graphs, a functional form is chosen for the parametric event studies to formally quantify the magnitude of the effects and their statistical significance.

The results suggest that the pandemic negatively affected employment, household income, migration, remittances, and food security. The probability of household heads losing jobs increased by seven percentage points in the first month of the pandemic and continued to increase further even after the sixth month of the pandemic. The household employment rate dropped by eleven percentage points, while the probability that no one in a household works immediately increased by nine percentage points in the first month of the pandemic. The loss of jobs has resulted in household incomes plummeting. The negative effects on wage incomes began to show up in the second month of the pandemic and exhibited some sign of recovery after the fifth month. Conversely, income from self-employment immediately declined by 36 percentage points in the first month and continued to worsen even after the sixth month. Remittances, an important income source for many households in Tajikistan, declined immediately with the start of the pandemic; however, by the end of the fifth month, they had bounced back to the pre-pandemic level. Nevertheless, no further increase in remittances above the pre-COVID level has been observed during the period of this study. While remittances show signs of recovery, migration has continued to fall since the beginning of the pandemic, and due to border closures and mobility restrictions has not shown signs of bouncing back. Although the results on migration and remittances are intuitive and consistent with the data, in this paper they are considered only indicative rather than causal due to their statistical insignificance.

Furthermore, the results suggest a worsening of food insecurity as rising unemployment and income loss could adversely affect the ability of households to afford food. Households immediately started eating an unhealthy diet with little diversity in the first months of the pandemic and then later ran out of food. In Tajikistan, the probability of households eating less than usual increased by three percentage points at the start of the pandemic, and those of eating unhealthy food with little diversity went up by 14 percentage points in the fourth month of the pandemic.

In the next step, I estimate heterogenous effects across location, pre-pandemic income levels, and household size. Urban households are more affected in terms of income and employment losses, while rural households are hit harder in terms of food security. Higher income and larger households are mostly self-employed and have lost employment and income due to declines in self-employment business. Lower income and smaller households also suffer in terms of lost income, however, not as large as the higher income and larger households. The lower income households are more prone to food insecurity.

Lastly, I estimate how households respond to the pandemic as an aggregate shock to household income. Unlike idiosyncratic shocks, risk sharing is difficult in the case of worldwide aggregate shocks. Due to the highly contagious nature of the COVID-19, households are also not able to use many coping mechanisms such as diversifying income sources, changing jobs, and moving to different places. My results show that households make use of three coping mechanisms in response to the pandemic brought shocks: borrowing, reducing food expenditure, and reducing health expenditure.

This paper contributes to the emerging literature on the impact of the COVID-19 pandemic in two ways. First, it provides direct causal effects on labor market outcomes, household income, and food security in a developing country setting where no lockdowns were implemented. Lockdowns have become a common Government response to manage the viral spread worldwide. By the end of March 2020, more than 100 countries implemented partial or full lockdowns and the number increased to 160 countries by December 2021 (Hale et al. 2021). Although lockdowns have found to be an effective measure against the spread of COVID-19 (Alfano & Ercolano, 2020), they come with social and economic costs (Gupta et al. 2020; Mahmud & Riley 2021; Martin et al. 2020; Rojas et al. 2020). On the other hand, a few countries^4^ have opted to more flexible approaches including non-legally binding state of emergencies and voluntary social restraints. Due to a lack of timely available data, most research on the socio-economic impacts of the absence of lockdown in the pandemic focuses on the cases of developed countries including Japan, Sweden, and South Korea. In the absence of government mandated lockdowns in South Korea, Aum et al. (2020) find that an increase in the confirmed cases leads to a fall in the rate of employment. In the case of Japan, Katafuchi et al (2021) find that non-legally binding COVID-19 policies reduce mobility through voluntary self-restraints and Kikuchi et al (2020) demonstrate that female, low-skilled, and non-flexible job holders are more adversely affected by COVID-19 income shocks even without mandated lockdowns. Similarly, Born et al (2021) show reduced mobility due to voluntary social restraints but less outcome loss than a lockdown would cause in Sweden.

Second, the paper provides estimates of heterogenous household effects and coping mechanisms adopted by households in addition to the causal impact of the pandemic. A number of studies have focused on developing countries and provided evidence on the impact of the pandemic on households but not on heterogeneous effects and coping strategies (Amare et al. 2020; Arndt et al. 2020; Kansiime et al. 2021). In the context of Tajikistan, a working paper by Shimizutani and Yamada (2021) analyzes how the pandemic affects various household welfare outcomes through its impact on migration and remittances and finds that remittances mitigate the negative effects of the pandemic on household welfare. My analysis adds to the existing studies with not only evidence of the dynamic causal effects of the pandemic directly on household income, labor market participation and food security, but also estimates of heterogenous household effects and household coping mechanisms in Tajikistan—a developing country that is susceptible to external shocks.

The remainder of the paper proceeds as follows. Section 2 briefly describes the COVID-19 pandemic situation and the containment policies implemented in Tajikistan. Section 3 discusses the data sources and introduces key variables. Section 4 explains the methodology and empirical approach. Section 5 presents the results and performs robustness checks to alternative specifications. Section 6 discusses the results in line with relevant literature and Section 7 concludes.

## The COVID-19 pandemic in Tajikistan

**Fig. 1:**
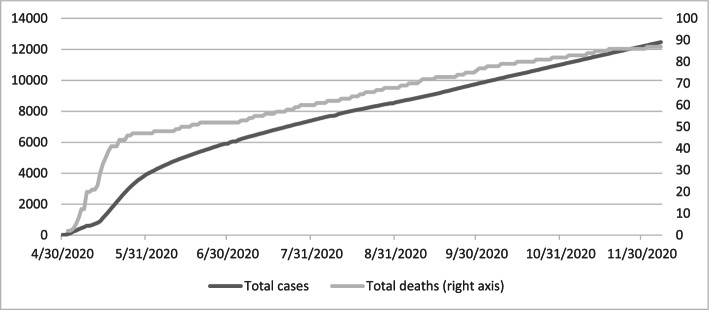
Total cumulative COVID-19 confirmed cases and deaths. Source: Author’s calculation based on Our World in Datahttps://ourworldindata.org/coronavirus/country/tajikistan?country=~TJK. (Accessed December 17, 2020.)

The Government of Tajikistan officially announced the country’s first 15 confirmed cases of COVID-19 in the capital city of Dushanbe and the second largest city, Khujand, in the northern province of Sughd on April 30, 2020. By May 7, all regions of the country had confirmed COVID-19 cases. Since the index cases, the number of confirmed cases continued to rise and by December 7, 2020, the total number of cases had reached 12,469 and the total number of deaths had reached 87 (Fig. 1).

**Fig. 2:**
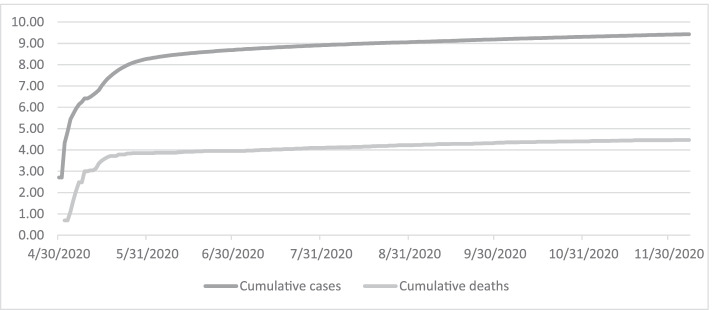
Logarithms of total cumulative COVID-19 confirmed cases and deaths, Source: Author’s calculation based on Our World in Data

From the early period of the pandemic in Tajikistan until the end of May 2020, the reproduction number, or the average number of people that one infected person will pass Sars-CoV-2 virus on to, was more than one; this indicated an exponential growth in the rate of spread. Since the end of May 2020, the reproduction number has gradually dropped to close to one and the rate of spread has slowed down as shown in Fig. 2. However, the total confirmed cases may not reflect the true spread of the COVID-19 disease as the testing capacity is limited in Tajikistan. According to the United Nations Tajikistan (2020), Tajikistan has only a few laboratories certified to conduct polymerase chain reaction (PCR) tests and limited testing kits constrain the number of tests performed daily. Additionally, the Government of Tajikistan stopped reporting the confirmed number of cases by region around mid-May and now only reports the national total of confirmed cases daily.

As part of the measures to contain the spread of the pandemic, the national and regional governments have introduced social distancing and measures to restrict mobility. As community transmission in Tajikistan was confirmed relatively late compared to neighboring countries, precautionary measures started even before its index cases. On March 20, Tajikistan suspended all international flights and closed its airport and borders due to concerns about the corona virus.^5^ Only a few border-crossing points with Uzbekistan and Kyrgyzstan were left open for international cargo.^6^ The national government announced the closure of kindergartens and schools between April 27 and May 10, which was later extended to August 16.

Although the country has not experienced a complete lockdown (United Nations 2020), the municipal governments have called upon citizens to voluntarily refrain from going out for non-urgent matters, particularly in large cities including Dushanbe and Khujand.^7^ By the end of April, theatres, cinemas, and public events had been cancelled. The Dushanbe municipal government closed beauty salons, hairdressers, and clothing markets for a month from April 30 to May 31. As working from home is limited in the country, the government agencies sent elderly workers and retirees on unpaid leave in mid-September for an indefinite period. In Dushanbe, 50% of the employees in institutions financed by the Dushanbe city budget were asked to go on unpaid leave for an indefinite period, with the exception of essential workers.^8^

Although the country did not mandate a full lockdown, partial measures such as shutting small businesses and services, releasing workers for unpaid leave, and calling for voluntary stay-at-home initiatives could disrupt economic activities. Given its highly informal economy, Tajikistan is particularly susceptible to economic shocks and resulting food insecurities. Additionally, employment is usually not guaranteed, and disruption brought about by the pandemic is likely to increase unemployment and reduce household incomes, which in turn results in food shortages and food insecurities.

## Data

The data used in this paper come from the Listening to Tajikistan (L2TJK) survey, an ongoing monthly household survey that has been administered through phone interviews since May 2015. The survey collects data on a variety of topics including household income, employment, migration, subjective wellbeing, and access to services from a nationally representative sample of 800 households from all regions in Tajikistan. The 800 households in the L2TJK sample represent a sub-sample of a nationally representative face-to-face survey of 3000 households conducted in spring 2015. The sampling weights have been adjusted for non-responses and refusals in every survey round to ensure the representativeness of the sample. In cases of attritions, replacement households have been selected from the same primary sampling units. Up to the end of November 2020 (the time of writing this paper), a total of 72 survey rounds had been conducted. Thus, the survey contains a rich set of panel data covering both pre- and post-COVID periods.

In addition to post-COVID rounds, this paper uses pre-COVID rounds covering approximately one year prior to the first confirmed COVID cases in Tajikistan for the purpose of pre- and post-comparisons. Furthermore, the pre-COVID period is restricted to one year prior to the pandemic in order to avoid any compounding effects that have happened outside the pandemic. The first confirmed COVID case in Tajikistan coincided with the beginning of Ramadan, the Islamic holy month celebrated widely in the country. Given this coincidence, this paper adopts the Islamic Lunar Calendar for pre- and post-COVID comparisons and set the pre-COVID period from May 1, 2019, the beginning of Ramadan in 2019. Therefore, the pre-COVID periods cover the period from May 2019 to March 2020, and the post-COVID period from April to November 2020. Consequently, the sample of this study comprises 19 rounds, of which 11 are pre-COVID and 8 post-COVID.

### Definitions of Outcome Variables

The L2TJK survey data contain information on the economic activities and subjective wellbeing of households over multiple rounds both before and after beginning of the COVID pandemic. Based on the available data, this paper focuses on the direct impact of the COVID-19 pandemic on the following set of output variables: (i) household income and employment; (ii) household migration and remittance statuses; and (iii) food security.

#### Household Income and Employment Variables

The L2TJK survey collects household incomes from various sources including wage-employment, self-employment, remittances, and pension and social welfare. The household total income is calculated by summing up incomes from all sources. For comparisons over time, all monetary values denominated in current prices of Somoni have been converted into the price from April 2015. Per capita income values are calculated by dividing the total incomes by household sizes.

In addition to income variables, the survey asks about the number of household members engaged in income-earning activities, such as wage-employment, self-employment, and the number of household members looking for a job. Based on these questions, two output variables are created: household employment rates and the indicator for no-one in a household working. The employment rate is calculated by dividing the number of employed household members by its economically active members, which in turn is computed by summing up employed and job-seeking household members. The indicator for no-one working is a binary variable that takes the value 1 if the household does not have a member engaged in employment activities.

#### Migration and Remittances

Migration and remittances are major contributing factors to Tajikistan’s economy and are likely to be hit hard by the COVID pandemic due to economic slowdowns and policy interventions in migrants’ destination countries. The L2TJK collects data on whether any household members have migrated abroad to work and whether they send remittances back home. Based on these questions, two binary variables for migration and remittance-receiving households are generated. In addition, data on monthly remittance amounts have been collected both before and after the pandemic. The monetary value of remittances is converted into April 2015 prices denominated in Somonis. Per capita remittances are calculated by dividing the total remittances by household sizes.

#### Food Security Indicators

The L2TJK survey collects data on several food security-related indicators over multiple rounds. This paper uses eight of these indicators that capture households’ experience of food insecurity. These indicators are self-reported experiences of food shortage and concerns about diet in the past month. More specifically, the survey collects data on food security by asking whether the households encountered a time in the past month (prior to the interview) when: (1) they worried about not having enough food; (2) they were unable to have healthy and nutritious food; (3) they were consuming only a few kinds of food; (4) they skipped a meal; (5) they ate less than usual; (6) they ran out of food; (7) they were hungry but did not eat; and (8) they went without eating for an entire day because of a lack of money and other resources. Based on these questions, binary variables are created for each of the 8 food-insecurity related indicators.

### Summary Statistics of Output variables by Pre- and post-COVID-19 Periods

Summary statistics of the main output variables are presented in Table 1. For comparison purposes, summary statistics are reported separately for the pre- and post-COVID periods. As stated, the pre-COVID period was between May 2019 and March 2020, and the post-COVID period was between April and November 2020.

**Table 1:** Summary statistics, pre- and post-COVID periods

	Pre-COVID		Post-COVID	
Variable	Mean	Std.Dev	Mean	Std.Dev
Total income	450.49	736.16	345.47	647.26
Remittance receipt	125.95	491.29	93.03	410.21
Wage income	97.55	313.88	77.37	274.18
Self-employment income	111.76	379.92	94.03	386.55
Pension income	54.27	139.62	50.93	132.93
Head of household is working	0.53	0.50	0.51	0.50
Employment rate	0.79	0.40	0.78	0.42
No-one works	0.20	0.40	0.22	0.42
Per capita income	73.65	123.22	57.21	109.06
Per capita remittances	19.94	80.55	14.75	63.20
Per capita wage income	18.32	60.70	14.65	54.29
Per capita self-employment income	19.25	66.11	15.91	62.99
Per capita pension income	9.88	28.78	9.34	27.07
Migrant household	0.33	0.47	0.32	0.47
Remittance receiving household	0.12	0.32	0.06	0.24
Worried about lack of food	0.43	0.50	0.42	0.49
Unhealthy food consumption	0.38	0.48	0.37	0.48
Low diversity food consumption	0.39	0.49	0.38	0.49
Skipped meal	0.24	0.43	0.24	0.43
Ate less	0.26	0.44	0.23	0.42
Food ran out	0.12	0.33	0.10	0.29
Went hungry	0.06	0.24	0.04	0.19
No food day	0.04	0.20	0.03	0.16
Observations	13,572		9,544	

Author's computations

Std.Dev = Standard deviation. The summary statistics are for pooled sample for pre- and post-COVID periods

Household income and employment have dropped considerably since the first COVID incidence. By sources of income, remittances have fallen by as much as 26% from their pre-COVID level. Domestic wage incomes and self-employment incomes have also declined by up to 15–20%. The declines in household incomes have resulted in sharp drops in per capita incomes, as the household size stayed relatively constant over the 19 rounds. Household employment rates have dropped by 1.3% while the number of households with no working members has increased drastically by 11%, compared to the same period before the COVID pandemic. The number of household heads working decreased by 3.2% from the pre-COVID period.

Due to border closures in Tajikistan and destination countries, many Tajik migrants are stranded at borders. Tajikistan closed all its borders on March 20, with only repatriation flights being operated.^9^ Russia, the main destination country for Tajik migrants, closed its borders in mid-March.^10^ While many seasonal migrant workers go to Russia in March–April every year to work, the timing of the border closures coincided with the seasonal migration of Tajik workers. However, the simple before and after comparison of the share of households with migrant household members shows only a small decrease in migration. Conversely, the number of remittance-receiving households has dropped sharply to 6% from the pre-COVID level of 12%, which may reflect the hardships faced by many migrants in their destination countries during the pandemic. At the same time, the relatively high migration rate during the pandemic could indicate the barriers to return migration due to the border closures.

Contrary to expectations, the food security indicators did not show any changes or showed only slight improvements after the first incidence of COVID-19. However, it should be noted that the beginning of each period corresponded with Ramadan. The share of households that skipped meals or consumed less food has not increased since the first incidence of COVID. This may reflect the fact that the President of Tajikistan called upon citizens to refrain from fasting during Ramadan to safeguard against infectious diseases amid the pandemic.^11^

## Methodology

### Non-parametric Event Study

The non-parametric event study is useful as a means of visually and flexibly assessing the pattern of outcomes relative to the “event” or the first confirmed community transmission case of COVID-19 in Tajikistan. The patterns of the outcomes are analyzed using the coefficients on various indicator variables for time relative to the “event”. The non-parametric event study takes the following form:

1 $${y}_{it}={\mu }_{i}+{\theta }_{t}+\sum_{j=\underline{j}}^{\overline{j}}{\beta }_{j}{b}_{it}^{j}+{\varepsilon }_{it}$$

where ${y}_{it}$ is the output variable of interest for households *i* interviewed during round (month) *t*, ${\mu }_{i}$ and ${\theta }_{t}$ are household and time (survey round or month) fixed effects, respectively. ${\varepsilon }_{it}$ is an idiosyncratic error term assumed to be uncorrelated with ${b}_{it}^{j}$ conditional on ${\mu }_{i}$ and ${\theta }_{t}$.

${b}_{it}^{j}$ is an event dummy and $j$ indexes survey rounds relative to the time of the COVID-19 index cases which is normalized to set to zero, that is $j=0$. To resolve any possible contamination issues that could arise from simultaneous events other than the COVID-19 incidence in a longer time span, the event window for this study is restricted to periods between approximately one year prior to and 11 months after the first COVID-19 incidence. In order to improve the identification, I bin the lags and leads of the “event” to $\underline{j}=-6$ and $\overline{j}=6$. The coefficients on the event dummies, ${\beta }_{j}$, represent the difference in outcome variables *j* in the month before or after the first COVID-19 cases relative to the reference period. Since Eq. (1) is a fully saturated model with all units treated,^12^ at least two relative period indicators need to be excluded to avoid multicollinearity (Borusyak & Jaravel, 2018). As excluding relative periods immediately preceding the “event” is common practice in the literature (Sun & Abraham 2020), two periods ($j=-2$ and $j=-1$) leading to the COVID-19 index cases are excluded to be served as the reference period. The coefficients on the endpoints, ${\beta }_{-6}$ and ${\beta }_{6}$ indicate whether a household is observed 6 or more periods before and after the “event”.

Equation (1) is a staggered adoption design where households are “treated” at different times because Tajikistan’s regions confirmed their first community transmission cases at different times. Dushanbe and Sughd were the first to confirm COVID-19 cases in April, while the rest of the country announced their first confirmed cases at various times in May. There may or may not be never treated units in an event study design. If there are never treated units, the event study design nests difference-in-difference design. In the case of Eq. (1), there are no never-treated units. All households are assumed to be subject to COVID-19 since the time their regions of residence announced their first COVID-19 cases. Even in the absence of never-treated households, the event dummy coefficients, ${\beta }_{j}$, can be interpreted as dynamic treatment effects relative to the reference period. Equation (1) is estimated by two-way fixed effects regressions and the treatment effects are allowed to vary over time non-parametrically. All analyses allow for an arbitrary variance–covariance matrix at the household level and include the relevant sample weights.

## Parametric event study

A parametric event study design is used to summarize the magnitude of the estimated treatment effects and their statistical significance. The choice of the functional form is guided by the patterns seen in the non-parametric event studies. The parametric event study design of this paper uses the following form:

2 $${y}_{it}={\mu }_{i}^{\prime}+{\theta }_{t}^{\prime}+\alpha {b}_{it}+\sum_{j=0}^{6}{\beta }_{j}{b}_{it}^{{\prime}j}+{\varepsilon }_{it}^{\prime}$$

where ${b}_{it}$ captures a linear pre-trend in event time and all other variables are the same as in Eq. (1). The coefficients of interest are ${\beta }_{j}$ that show the changes in the outcome following the index case of COVID-19 relative to any pre-existing linear trend, $\alpha$.

As before, Eq. (2) is estimated by two-way fixed effects regressions and includes the appropriate sample weights. The coefficients, ${\beta }_{j}$, are interpreted as dynamic treatment effects of the COVID-19 pandemic.

## Results and Discussions

### Main Results

Graphical analyses based on the non-parametric event study are presented first. The ${\beta }_{j}$ coefficients and the corresponding 95% confidence intervals of the non-parametrical event study model given by Eq. (1) are plotted against periods relative to the timing of the COVID-19 index cases. Since the L2TJK survey is conducted monthly, the relative periods can be interpreted as the relative months to the month of the survey round when the COVID-19 index cases are confirmed.

**Fig. 3:**
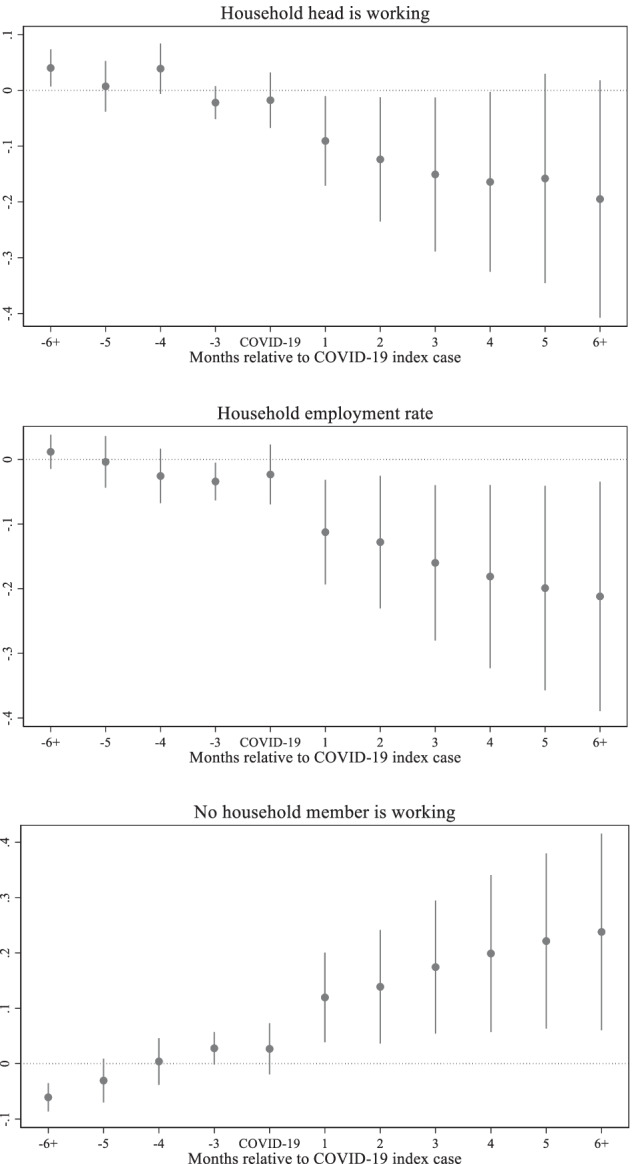
Effects of COVID-19 on household employment outcomes, Source: Author's estimations, Note: The base period is 1 period preceding the COVID-19 index case. The points in each figure represent the estimated effects of event time, that is, *β*_*j*_ coefficients from the non-parametric event study model (1), relative to the COVID-19 index cases. The straight lines represent 95% confidence intervals

Figure 3 depicts the dynamic effects of COVID-19 on household employment outcomes: the employment status of household heads, household employment rates, and share of households where no member is engaged in employment activities. The first two panels in Fig. 3 show that there is a clear declining trend in both the employment status of household heads and household employment rates. The last panel shows that the share of households with no-one working has increased relative to the reference period. For all three indicators there is a clear linear pre-trend.

**Fig. 4:**
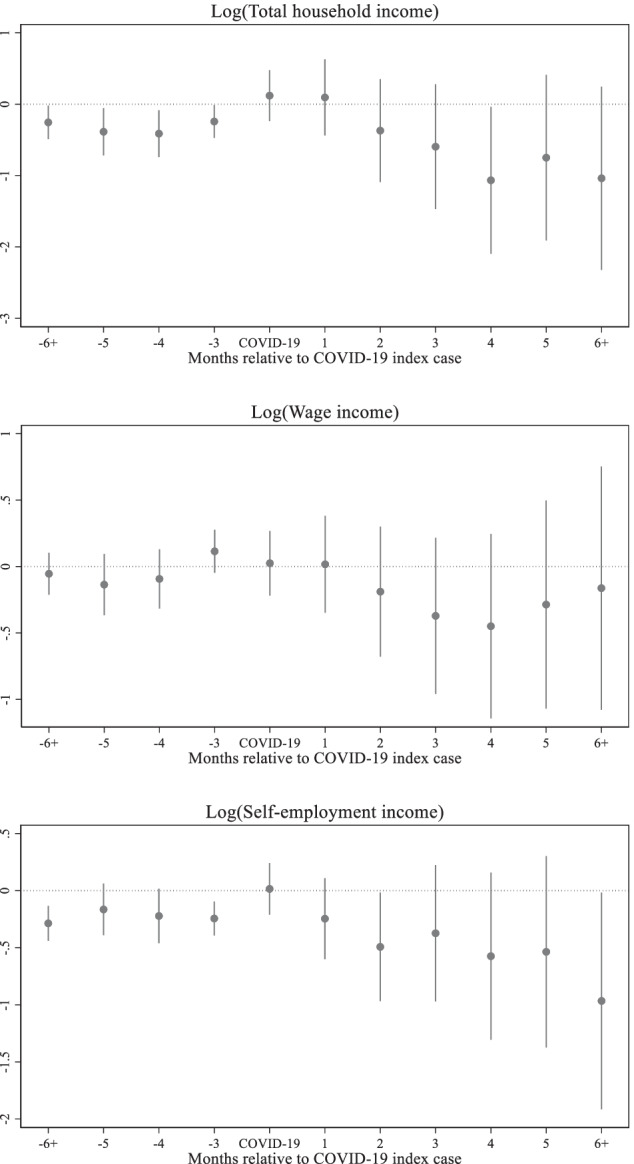
Effects of COVID-19 on household total incomes, Source: Author's estimations, Note: The base period is 1 period preceding the COVID-19 index case. The points in each figure represent the estimated effects of event time, that is, *β*_*j*_ coefficients from the non-parametric event study model (1), relative to the COVID-19 index cases. The straight lines represent 95% confidence intervals

Figure 4 shows the dynamic effect of COVID-19 on household total incomes by source. The negative effect on wage incomes kicked in two months after the confirmation of the first COVID-19 cases in each region. The wage income continued to drop until the fourth month after the COVID-19 index cases and started to pick up from the fifth month. However, the level of wage income remains well below the level of the reference period even after the sixth month. The effect on the total income follows the pattern of the effect on the wage income until the fifth month after the COVID-19 index cases. From the sixth month, the effect on total income is likely to be governed by the effect of the self-employment income. Unlike the wage income, the effect on the self-employment income is immediately observed in the first month after the COVID-19 index cases. While some recovery can be seen in the third and fifth months, the general trend is decline over time.

**Fig. 5:**
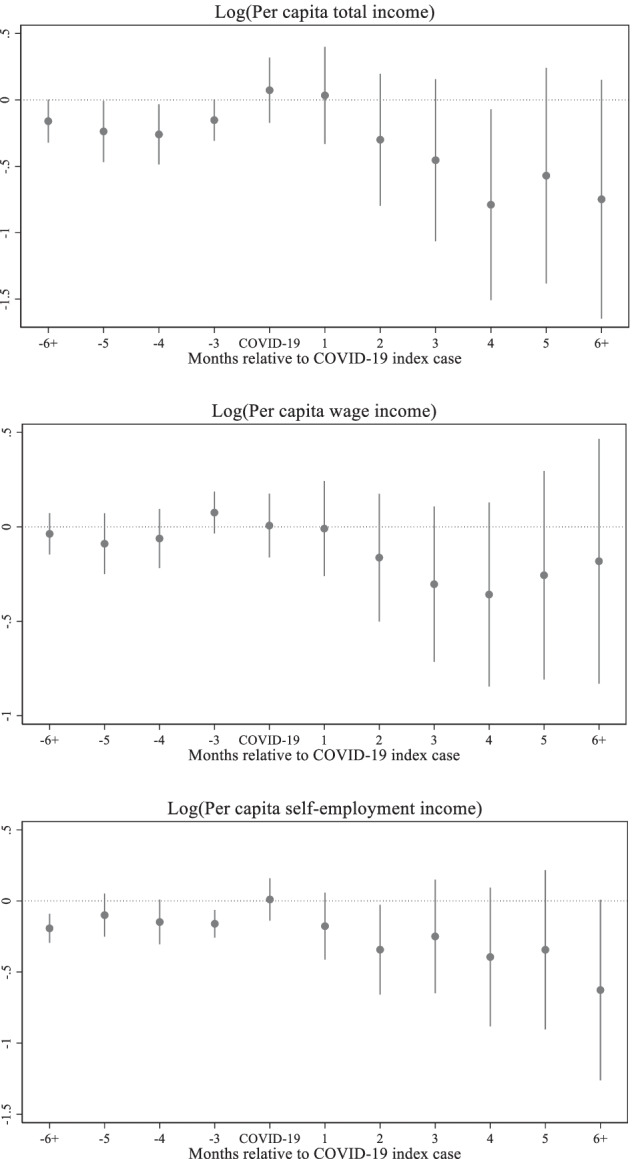
Effects of COVID-19 on household per capita incomes, Source: Author's estimations, Note: The base period is 1 period preceding the COVID-19 index case. The points in each figure represent the estimated effects of event time, that is, *β*_*j*_ coefficients from the non-parametric event study model (1), relative to the COVID-19 index cases. The straight lines represent 95% confidence intervals

The effects on per capita income follow the same pattern as the total income. As shown in Fig. 5, per capita self-employment income immediately declines after the first confirmed case, while per capita wage income and per capita total income start to fall from the second month. The per capita wage income shows some sign of recovery from the fifth month, although the per capita self-employment income further falls even after the sixth month.

**Fig. 6:**
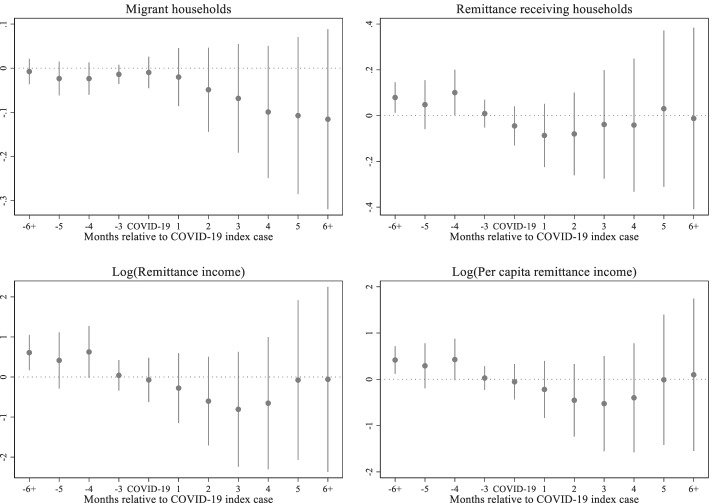
Effects of COVID-19 on household migration and remittance outcomes, Source: Author's estimations, Note: The base period is 1 period preceding the COVID-19 index case. The points in each figure represent the estimated effects of event time, that is, *β*_*j*_ coefficients from the non-parametric event study model (1), relative to the COVID-19 index cases. The straight lines represent 95% confidence intervals

Figure 6 shows that migration and remittances start to fall immediately after the COVID-19 index cases. In the case of migration, they continue to drop even 6 months after the index cases. In contrast, remittances show signs of recovery around the fifth month after the COVID-19 index cases and reach approximately the pre-COVID level in the sixth month and onwards. The pattern is the same for all three remittance indicators.

**Fig. 7:**
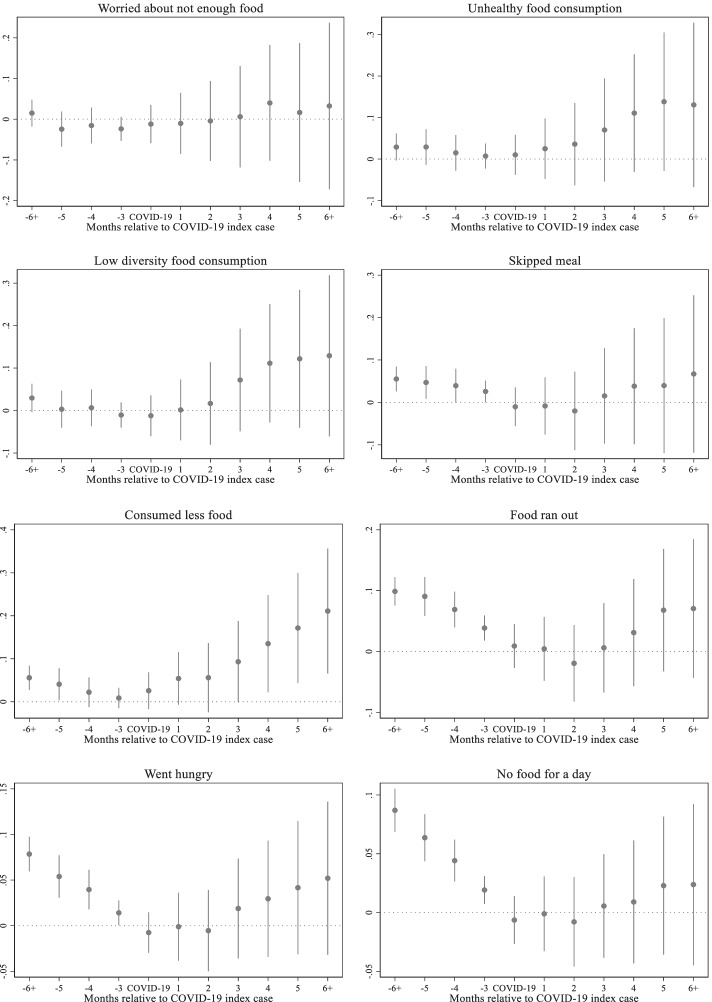
Effects of COVID-19 on household food security outcomes, Source: Author's estimations, Note: The base period is 1 period preceding the COVID-19 index case. The points in each figure represent the estimated effects of event time, that is, *β*_*j*_ coefficients from the non-parametric event study model (1), relative to the COVID-19 index cases. The straight lines represent 95% confidence intervals

The results for food security indicators are presented in Fig. 7. While some of the food security indicators should be interpreted with caution due to the overlap with the Ramadan period, the results generally indicate that COVID-19 increases food insecurity. Compared to the reference period, more households consider themselves to be consuming unhealthy and low diversity diets. While the number of households worried about not having enough food due to a lack of finances and other resources has not increased compared to the reference period, the number of households consuming less food than usual increased immediately after the COVID-19 index cases. The number of households that skipped a meal, ran out of food, went hungry and/or had no food for a whole day increased from the third and fourth months after the COVID-19 index cases.

After graphically visualizing the dynamic effects of the COVID-19 based on the non-parametrical event study design, I use the parametric event study design to formally summarize the magnitude of the effects and their statistical significance. If the coefficients on the “event” dummies after the confirmed COVID-19 index cases are to be interpreted as the causal effect of COVID-19, the timing of COVID-19 index cases would need to be uncorrelated with the outcome variables. This implies that there would be no trend in the outcomes leading up to the “event”. However, this is a strong assumption that cannot be supported by data. The parametric event studies require a weaker identifying assumption that the timing of the COVID-19 index cases is not correlated with deviations from the outcome’s linear trend in event time. Thus, based on the pre-trend patterns seen in the non-parametric event studies, the functional form chosen for the parametrical event studies allows a linear pre-trend as in Eq. (2). The parametrical event studies given by Eq. (2) are estimated using two-way fixed effects regressions.

Table 2 presents the estimation results for household employment outcomes. The results are consistent with the non-parametric studies and show the significant negative effects that COVID-19 has on household employment outcomes. The probability of household heads being employed and household employment rates immediately decreased with the confirmed COVID-19 index cases. However, statistically significant effects start to prevail around one month into the pandemic. While the statistically significant negative impact of COVID-19 continued for the first two months in relation to the employment status of household heads, it persisted for the households’ employment rate even after the sixth month. At the end of the fifth month after the COVID-19 index cases were confirmed, household employment rates dropped by 18 percentage points and continued to fall to 19 percentage points after the sixth month. At the same time, the probability of households with no-one engaged in employment activities also immediately increased after the COVID-19 index cases. In the first month after the COVID-19 index cases were confirmed, the share of households with no-one working increased by 9.2 percentage points. Although the effect becomes statistically insignificant around the sixth month and beyond, the trend continues to increase.

**Table 2:** Impact of COVID-19 on household's employment outcomes

	(1)	(2)	(3)
VARIABLES	Head is working	Employment rate	No-one works
Pre-COVID trend	-0.0104***	-0.00370	0.0153***
	(0.00390)	(0.00306)	(0.00294)
COVID-19	-0.00822	-0.0201	0.0127
	(0.0254)	(0.0237)	(0.0236)
1-month effect	-0.0717*	-0.106**	0.0915**
	(0.0413)	(0.0413)	(0.0413)
2-month effect	-0.0952*	-0.118**	0.0967*
	(0.0574)	(0.0526)	(0.0526)
3-month effect	-0.113	-0.147**	0.118*
	(0.0711)	(0.0619)	(0.0619)
4-month effect	-0.117	-0.165**	0.129*
	(0.0834)	(0.0732)	(0.0732)
5-month effect	-0.101	-0.180**	0.137*
	(0.0972)	(0.0820)	(0.0820)
6 plus-month effect	-0.128	-0.190**	0.139
	(0.110)	(0.0920)	(0.0920)
Observations	23,053	23,053	23,053
R-squared	0.478	0.383	0.389
Household fixed effects	Yes	Yes	Yes
Round fixed effects	Yes	Yes	Yes

Author's estimations

Robust standard errors in parentheses. *** *p* < 0.01, ** *p* < 0.05, * *p* < 0.1

The dynamic impacts of COVID-19 on household incomes are presented in Table 3. Consistent with the graphical analyses, all income sources decline after the first COVID-19 cases were confirmed. Compared to the reference period, total household income decreases by almost 55% in the third month and continues to decline even after the sixth month in the aftermath of the first confirmed cases. This significant decline seems to be driven mostly by the reductions in incomes from self-employment, which immediately suffered a 30% fall in the first month after the COVID-19 index cases. Although statistically insignificant, wage income is also negatively affected through-out the periods after the “event” and shows some recovery at the end of the fifth month. The results for per capita income follow the same pattern as the total household income, although the magnitude is somewhat smaller; this indicates that large households could be hit disproportionately hard.

**Table 3:** Impact of COVID-19 on household income

	(1)	(2)	(3)	(4)	(5)	(6)
VARIABLES	Total income	Wage income	Self-employment income	Per capita total income	Per capita wage income	Per capita self-employment income
Pre-COVID trend	0.0570**	0.0191	0.0572***	0.0363*	0.0126	0.0389***
	(0.0277)	(0.0186)	(0.0179)	(0.0192)	(0.0129)	(0.0119)
COVID-19	0.0649	0.00753	-0.0409	0.0379	-0.00479	-0.0274
	(0.180)	(0.122)	(0.114)	(0.124)	(0.0848)	(0.0751)
1-month effect	-0.0153	-0.0174	-0.358**	-0.0362	-0.0322	-0.253**
	(0.270)	(0.183)	(0.179)	(0.185)	(0.126)	(0.119)
2-month effect	-0.536	-0.241	-0.660***	-0.405	-0.197	-0.457***
	(0.366)	(0.247)	(0.240)	(0.253)	(0.172)	(0.159)
3-month effect	-0.816*	-0.440	-0.597**	-0.594*	-0.349*	-0.401**
	(0.444)	(0.299)	(0.303)	(0.311)	(0.210)	(0.202)
4-month effect	-1.342**	-0.535	-0.854**	-0.964***	-0.415*	-0.584**
	(0.524)	(0.357)	(0.372)	(0.366)	(0.251)	(0.248)
5-month effect	-1.080*	-0.389	-0.872**	-0.780*	-0.325	-0.571**
	(0.594)	(0.405)	(0.425)	(0.416)	(0.286)	(0.283)
6 plus-month effect	-1.423**	-0.282	-1.358***	-0.993**	-0.262	-0.892***
	(0.658)	(0.476)	(0.484)	(0.462)	(0.337)	(0.323)
Observations	23,053	23,053	23,053	23,053	23,053	23,053
Household FE	Yes	Yes	Yes	Yes	Yes	Yes
Round FE	Yes	Yes	Yes	Yes	Yes	Yes

Author's estimations

Robust standard errors in parentheses. *** *p* < 0.01, ** *p* < 0.05, * *p* < 0.1. Dependent variables are in logs

Results for the household migration and remittance outcomes are displayed in Table 4. While the pattern of the impacts is consistent with the graphical analyses, the results are not statistically significant.

**Table 4:** Impact of COVID-19 on household migration and remittance outcomes

	(1)	(2)	(3)	(4)
VARIABLES	Migrant household	Remittance-receiving household	Remittance receipt	Per capita remittances
Pre-COVID trend	0.00184	-0.0192**	-0.148***	-0.101***
	(0.00344)	(0.00809)	(0.0537)	(0.0365)
COVID-19	-0.0115	-0.0275	0.0612	0.0371
	(0.0182)	(0.0431)	(0.277)	(0.193)
1-month effect	-0.0237	-0.0518	-0.00881	-0.0356
	(0.0335)	(0.0710)	(0.443)	(0.313)
2-month effect	-0.0542	-0.0275	-0.199	-0.178
	(0.0484)	(0.0929)	(0.564)	(0.400)
3-month effect	-0.0756	0.0316	-0.268	-0.157
	(0.0624)	(0.121)	(0.730)	(0.523)
4-month effect	-0.108	0.0466	0.0206	0.0606
	(0.0759)	(0.149)	(0.837)	(0.599)
5-month effect	-0.118	0.136	0.734	0.543
	(0.0902)	(0.175)	(1.010)	(0.715)
6 plus-month effect	-0.128	0.111	0.888	0.744
	(0.103)	(0.202)	(1.164)	(0.832)
Observations	23,053	7,529	7,529	7,529
Household FE	Yes	Yes	Yes	Yes
Round FE	Yes	Yes	Yes	Yes

Author's estimations

Robust standard errors in parentheses. *** *p* < 0.01, ** *p* < 0.05, * *p* < 0.1. Dependent variables in columns 3 and 4 are in logs. The sample size for models (2)-(4) is restricted to migrant households only

Table 5 shows the impacts of COVID-19 on household food security indicators. The probability of households eating less, running out of food, going hungry, and having no food for a day immediately increased in the aftermath of the COVID-19 index cases. Subsequently, households who consider themselves to be eating an unhealthy and low diversity diet have been increasing gradually since around the fourth month into the “event”. Around the sixth month and beyond, the probability of households eating unhealthy and low diversity food increased by 17.6 percentage points, while households eating less and running out of food due to a lack of resources increased by 30 and 21 percentage points, respectively. Similarly, the number of households that ran out of food and had no food for a day has increased up to 17.3 and 15.8 percentage points, respectively. Although food security indicators measured in availability and quality of food suggest worsening food security among Tajik households, the number of households that worry about not having enough food have not increased significantly.

**Table 5:** Impact of COVID-19 on household food security outcomes

	(1)	(2)	(3)	(4)	(5)	(6)	(7)	(8)
VARIABLES	Worried about not enough food	Unhealthy food consumption	Low diversity food consumption	Skipped meal	Ate less	Food ran out	Went hungry	No food for a day
Pre-COVID trend	-0.00419	-0.00692*	-0.00713*	-0.0132***	-0.0137***	-0.0223***	-0.0185***	-0.0204***
	(0.00394)	(0.00381)	(0.00397)	(0.00347)	(0.00330)	(0.00276)	(0.00223)	(0.00217)
COVID-19	-0.00833	0.0168	-0.00551	0.00200	0.0384*	0.0302*	0.00976	0.0127
	(0.0239)	(0.0244)	(0.0246)	(0.0231)	(0.0218)	(0.0181)	(0.0113)	(0.0102)
1-month effect	-0.00301	0.0380	0.0148	0.0162	0.0794**	0.0467*	0.0335*	0.0371**
	(0.0379)	(0.0370)	(0.0363)	(0.0341)	(0.0310)	(0.0265)	(0.0189)	(0.0159)
2-month effect	0.00652	0.0555	0.0366	0.0167	0.0939**	0.0442	0.0465**	0.0493**
	(0.0496)	(0.0503)	(0.0490)	(0.0467)	(0.0410)	(0.0317)	(0.0226)	(0.0192)
3-month effect	0.0206	0.0960	0.0984	0.0644	0.144***	0.0910**	0.0881***	0.0819***
	(0.0625)	(0.0629)	(0.0604)	(0.0565)	(0.0477)	(0.0371)	(0.0278)	(0.0224)
4-month effect	0.0581	0.143**	0.145**	0.0997	0.198***	0.137***	0.116***	0.104***
	(0.0716)	(0.0714)	(0.0696)	(0.0685)	(0.0572)	(0.0441)	(0.0325)	(0.0267)
5-month effect	0.0382	0.177**	0.162**	0.113	0.247***	0.195***	0.146***	0.137***
	(0.0860)	(0.0844)	(0.0811)	(0.0798)	(0.0645)	(0.0500)	(0.0373)	(0.0303)
6 plus-month effect	0.0580	0.176*	0.176*	0.153	0.300***	0.219***	0.173***	0.158***
	(0.103)	(0.100)	(0.0946)	(0.0930)	(0.0732)	(0.0570)	(0.0426)	(0.0354)
Observations	23,053	23,053	23,053	23,053	23,053	23,053	23,053	23,053
R-squared	0.476	0.475	0.473	0.453	0.508	0.356	0.261	0.269
Household FE	Yes	Yes	Yes	Yes	Yes	Yes	Yes	Yes
Round FE	Yes	Yes	Yes	Yes	Yes	Yes	Yes	Yes

Author's estimations

Robust standard errors in parentheses. *** *p* < 0.01, ** *p* < 0.05, * *p* < 0.1

### Heterogenous Impacts

This subsection implements heterogenous household effects to detect how different types of households are affected by the pandemic. Given the available data, it is possible to conduct the heterogenous household effect analysis in terms of location, pre-pandemic per capita income level, and household size. For each type of households, the non-parametric event study model was estimated to visualize how the outcome variables are changing after the outbreak of the pandemic.

**Fig. 8:**
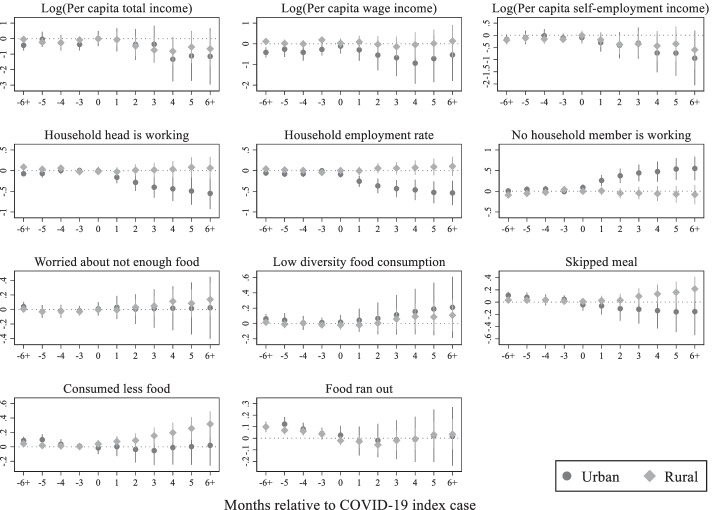
COVID-19 impacts by location, Source: Author's estimations, Note: The base period is 1 period preceding the COVID-19 index case. The points in each figure represent the estimated effects of event time, that is, *β*_*j*_ coefficients from the non-parametric event study model (1), relative to the COVID-19 index cases. The straight lines represent 95% confidence intervals

Figure 8 displays the heterogenous effects of the pandemic by households’ location: urban or rural. Results show that urban households are more likely to be affected in terms of lost income and employment, particularly wage employment. Conversely, rural household income is likely to be deteriorating to due the loss in self-employment income. While both urban and rural households are not completely running out of food, both reduced the diversity of food consumption amid the pandemic. Generally, rural households are found to be more prone to food insecurity. Although most rural households are engaged in agricultural self-employment; higher poverty, inefficient agricultural production, food price spikes, limited access to agricultural inputs are contributing to the high food insecurity in rural areas (United Nations 2020).

**Fig. 9:**
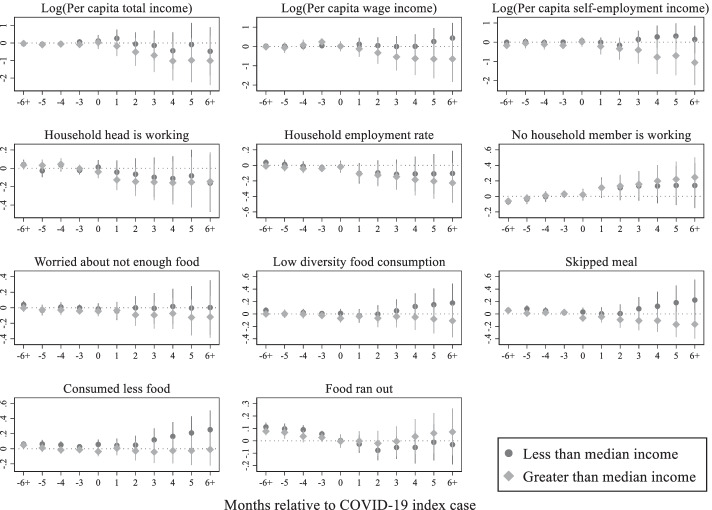
COVID-19 impacts by household per capita income, Source: Author's estimations, Note: The base period is 1 period preceding the COVID-19 index case. The points in each figure represent the estimated effects of event time, that is, *β*_*j*_ coefficients from the non-parametric event study model (1), relative to the COVID-19 index cases. The straight lines represent 95% confidence intervals

Differential impacts of the pandemic by pre-pandemic per capita household income are presented in Fig. 9. The sample is split into lower and higher than pre-pandemic median per capita income groups to see how income level affects the pandemic effects. Results show that while both types of households are equally affected by job losses, higher income households have lost employment and income to a larger extent than lower income households. Although the lost income in absolute terms is lower for low-income households, the lost income has greater implications for them than higher income households, making them more prone to food insecurity.

**Fig. 10:**
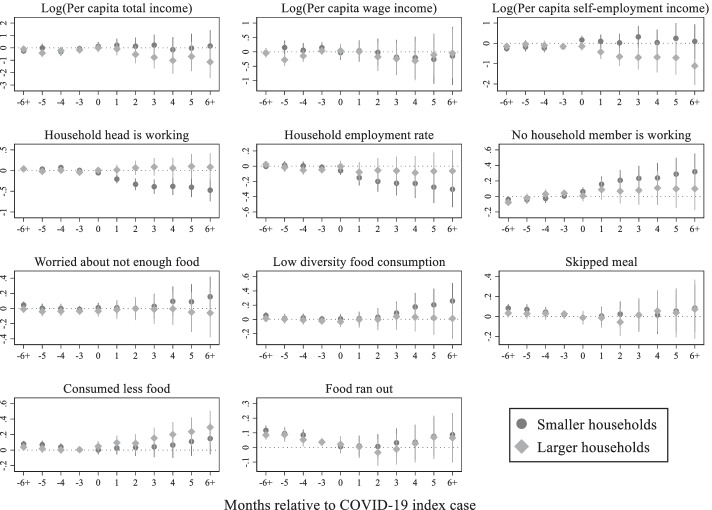
COVID-19 impacts by household size, Source: Author's estimations, Note: The base period is 1 period preceding the COVID-19 index case. The points in each figure represent the estimated effects of event time, that is, *β*_*j*_ coefficients from the non-parametric event study model (1), relative to the COVID-19 index cases. The straight lines represent 95% confidence intervals

Lastly, household heterogenous effects are analyzed by pre-pandemic household size in Fig. 10. Per capita income declined to a greater extent for larger households, mostly driven by the loss in self-employment income. On the other hand, smaller households are likely to be engaged in wage employment. Both large and small households are affected adversely in terms of food security.

### Coping Mechanisms

The COVID-19 pandemic has hit households as a worldwide aggregate shock that is difficult to be insured or shared within a wider community. Due to the highly contagious nature of the SARS-Cov-2 virus, households are also limited in their ability to diversify risks associated with the aggregate shock as changing jobs and locations are restricted during the pandemic. This leaves households with fewer options to smooth consumption when faced with the pandemic. This paper considers such coping strategies including dissaving, borrowing, selling assets, and reducing food and health expenses. To quantify which coping mechanism is used amid the pandemic, the following simple model is estimated.

3 $${coping}_{it}={\alpha }_{i}+{\beta }_{1}{COVID}_{it}+{\beta }_{2}{Z}_{it}+{e}_{it}$$

where ${coping}_{it}$ is a coping strategy (dissaving, borrowing, selling assets, and reducing food and health expenses) that household *i* adopted at time *t*, ${COVID}_{it}$ is an indicator variable whether a household *i* is subject the COVID-19 pandemic at time *t*, ${Z}_{it}$ is a vector of covariates (household size, whether household head is working, whether household members involuntarily reduced work, household employment rate, and dummy variable for higher than median income) that may affect the household’s decision to opt a coping mechanism. ${\alpha }_{i}$ is household fixed effects, ${\beta }_{1}$, and ${\beta }_{2}$ are parameters to be estimated and ${e}_{it}$ is an idiosyncratic error term. Equation (3) is estimated using a household fixed effects model to control for time invariant unobserved heterogeneities. The results are provided in Table 6.

**Table 6:** Coping mechanisms against the pandemic

	(1)	(2)	(3)	(4)	(5)
VARIABLES	Dissaving	Borrowing	Selling assets	Reducing food expenses	Reducing health expenses
COVID-19	0.008	0.023*	-0.009	0.136***	0.028***
	(0.007)	(0.011	(0.006	(0.011)	(0.011)
Household size	-0.042*	-0.033**	0.022	0.041**	0.002
	(0.022)	(0.014)	(0.014)	(0.016)	(0.009)
Head's work	0.029***	-0.017**	-0.004	-0.029***	-0.011
	(0.009)	(0.008)	(0.005)	(0.009)	(0.008)
Members lost job	0.027	0.059	-0.013	-0.015	0.032
	(0.046)	(0.052)	(0.024)	(0.043)	(0.053)
Members reduced work	-0.048	0.046	-0.098***	-0.049	-0.038
	(0.211)	(0.105)	(0.031)	(0.139)	(0.165)
Employment rate	-0.041***	-0.022**	-0.012*	-0.049***	0.015
	(0.01)	(0.009)	(0.006)	(0.01)	(0.001)
Higher than median income	0.044***	-0.007	0.044***	8.52e-05	-0.026***
	(0.007)	(0.006)	(0.005)	(0.006)	(0.006)
Observations	13,340	20,714	20,714	20,714	23,116
Household FE	Yes	Yes	Yes	Yes	Yes

Author's estimations

Robust standard errors in parentheses. *** *p* < 0.01, ** *p* < 0.05, * *p* < 0.1

Column (1) is only for households with savings. In Columns (2)-(4), the dependent variables are not observed in waves 68–69

The results show that households increased borrowing and reduced food and health expenses when faced with the COVID-19 pandemic. The probability of households reducing food expenses is likely to increase by 13.6 percentage points, while that of reducing health expenses is to increase by 2.8 percentage points. The probability of borrowing increases by 2.3 percentage points. Although statistically insignificant, households could be depleting their savings. On the other hand, households are unlikely to sell assets. This may indicate either households may not be able to sell assets amid the pandemic or they are reluctant to sell assets for their long-run consumption.

### Robustness Checks

This sub-section performs the following two specifications to investigate the sensitivity to alternative specifications of the parametric event study. The first alternative specification takes the following form:

4 $${y}_{it}={\mu }_{i}^{\prime}+{\theta }_{t}^{\prime}+\alpha {b}_{it}+\sum_{j=0}^{6}{\beta }_{j}{b}_{it}^{{\prime}j}+\gamma {X}_{it}+{\varepsilon }_{it}^{\prime}$$

where Eq. (2) is augmented by additional time-varying covariates, ${X}_{it}$. However, changes in these covariates should not be correlated with the timing of the “event”. Equation (3) includes household size as an additional covariate, because it is time-varying and assumed to be not correlated with the timing of the COVID-19 index cases.

The second alternative specification includes regional fixed effects instead of household fixed effects:

5 $${y}_{it}={\delta }_{i}^{\prime}+{\theta }_{t}^{\prime}+\alpha {b}_{it}+\sum_{j=0}^{6}{\beta }_{j}{b}_{it}^{{\prime}j}+{\varepsilon }_{it}^{\prime}$$

where ${\delta }_{i}^{\prime}$ is region fixed effects and $i$ indexes region. The remaining covariates are the same as before.

The estimation results of the two alternative specifications are presented in Tables 7, 8, and 9. Panel A shows the first alternative specification with an additional covariate, and Panel B shows the second alternative specification with regional fixed effects instead of household fixed effects. The results are generally reassuring and consistent with the main results. The sensitivity of the results for migration and remittances are not investigates, because the main results are not statistically significant for all outcomes related to migration and remittances.

**Table 7:** Robustness to alternative specifications for the household employment outcomes

	(1)	(2)	(3)
VARIABLES	Head is working	Employment rate	No-one works
*Panel A. Additional covariates*			
Pre-COVID trend	-0.0104***	-0.00369	0.0153***
	(0.00390)	(0.00306)	(0.00294)
COVID-19	-0.00811	-0.0200	0.0126
	(0.0254)	(0.0237)	(0.0236)
1-month effect	-0.0715*	-0.106**	0.0913**
	(0.0413)	(0.0413)	(0.0413)
2-month effect	-0.0949*	-0.118**	0.0964*
	(0.0574)	(0.0526)	(0.0526)
3-month effect	-0.112	-0.147**	0.118*
	(0.0711)	(0.0619)	(0.0619)
4-month effect	-0.116	-0.165**	0.129*
	(0.0834)	(0.0732)	(0.0733)
5-month effect	-0.0998	-0.179**	0.136*
	(0.0972)	(0.0820)	(0.0820)
6 plus-month effect	-0.128	-0.189**	0.139
	(0.110)	(0.0920)	(0.0920)
*Panel B. Regional fixed effects*			
Pre-COVID trend	-0.0108**	-0.00463	0.0159***
	(0.00449)	(0.00329)	(0.00324)
COVID-19	-0.00430	-0.0179	0.00959
	(0.0341)	(0.0278)	(0.0278)
1-month effect	-0.0595	-0.0966**	0.0809*
	(0.0488)	(0.0438)	(0.0438)
2-month effect	-0.0818	-0.110**	0.0869
	(0.0629)	(0.0543)	(0.0543)
3-month effect	-0.0767	-0.130**	0.0993
	(0.0756)	(0.0632)	(0.0632)
4-month effect	-0.0740	-0.143**	0.105
	(0.0884)	(0.0725)	(0.0725)
5-month effect	-0.0493	-0.152*	0.108
	(0.101)	(0.0820)	(0.0819)
6 plus-month effect	-0.0987	-0.160*	0.109
	(0.115)	(0.0917)	(0.0916)
Observations	23,116	23,116	23,116
Household FE	Yes	Yes	Yes
Round FE	Yes	Yes	Yes

Author's estimations

Robust standard errors in parentheses. *** *p* < 0.01, ** *p* < 0.05, * *p* < 0.1

**Table 8:** Robustness to alternative specifications for the household incomes

	(1)	(2)	(3)	(4)	(5)	(6)
VARIABLES	Total income	Wage income	Self-employment income	Per capita total income	Per capita wage income	Per capita self-employment income
*Panel A. Additional covariates*						
Pre-COVID trend	0.0569**	0.0191	0.0571***	0.0363*	0.0126	0.0388***
	(0.0277)	(0.019)	(0.0179)	(0.0192)	(0.0129)	(0.0119)
COVID-19	0.0643	0.00721	-0.0417	0.0375	-0.00505	-0.0280
	(0.180)	(0.122)	(0.114)	(0.124)	(0.0848)	(0.0751)
1-month effect	-0.0161	-0.0180	-0.359**	-0.0369	-0.0326	-0.254**
	(0.270)	(0.183)	(0.179)	(0.185)	(0.126)	(0.119)
2-month effect	-0.537	-0.242	-0.663***	-0.406	-0.198	-0.458***
	(0.366)	(0.247)	(0.240)	(0.253)	(0.172)	(0.159)
3-month effect	-0.818*	-0.441	-0.601**	-0.595*	-0.350*	-0.403**
	(0.444)	(0.299)	(0.303)	(0.311)	(0.210)	(0.202)
4-month effect	-1.342**	-0.535	-0.856**	-0.964***	-0.416*	-0.585**
	(0.524)	(0.357)	(0.371)	(0.366)	(0.251)	(0.247)
5-month effect	-1.084*	-0.392	-0.879**	-0.784*	-0.327	-0.576**
	(0.594)	(0.405)	(0.424)	(0.416)	(0.286)	(0.283)
6 plus-month effect	-1.424**	-0.283	-1.359***	-0.993**	-0.262	-0.893***
	(0.659)	(0.476)	(0.483)	(0.462)	(0.337)	(0.322)
*Panel B. Regional fixed effects*						
Pre-COVID trend	0.0627**	0.0181	0.0586***	0.0417**	0.0126	0.0393***
	(0.0287)	(0.019)	(0.0198)	(0.0197)	(0.0131)	(0.0131)
COVID-19	0.0511	0.0336	-0.0773	0.0255	0.0125	-0.0603
	(0.211)	(0.146)	(0.149)	(0.144)	(0.101)	(0.0967)
1-month effect	-0.0468	0.00830	-0.403*	-0.0543	-0.0145	-0.292**
	(0.305)	(0.206)	(0.208)	(0.209)	(0.143)	(0.137)
2-month effect	-0.555	-0.184	-0.729***	-0.399	-0.156	-0.507***
	(0.393)	(0.257)	(0.276)	(0.269)	(0.179)	(0.182)
3-month effect	-0.857*	-0.367	-0.634*	-0.596*	-0.299	-0.429*
	(0.477)	(0.309)	(0.347)	(0.328)	(0.215)	(0.229)
4-month effect	-1.395**	-0.478	-0.869**	-0.978**	-0.379	-0.596**
	(0.558)	(0.357)	(0.414)	(0.383)	(0.248)	(0.273)
5-month effect	-1.172*	-0.305	-0.908*	-0.815*	-0.271	-0.597*
	(0.641)	(0.408)	(0.480)	(0.442)	(0.284)	(0.319)
6 plus-month effect	-1.537**	-0.243	-1.408**	-1.052**	-0.242	-0.927**
	(0.725)	(0.455)	(0.548)	(0.501)	(0.317)	(0.364)
Observations	23,116	23,116	23,116	23,116	23,116	23,116
Household FE	Yes	Yes	Yes	Yes	Yes	Yes
Round FE	Yes	Yes	Yes	Yes	Yes	Yes

Author's estimations

Robust standard errors in parentheses. *** *p* < 0.01, ** *p* < 0.05, * *p* < 0.1

**Table 9:** Robustness to alternative specifications for the household food security outcomes

	(1)	(2)	(3)	(4)	(5)	(6)	(7)	(8)
VARIABLES	Worried about not enough food	Unhealthy food consumption	Low diversity food consumption	Skipped meal	Ate less	Food ran out	Went hungry	No food for a day
*Panel A. Additional covariates*								
Pre-COVID trend	-0.00419	-0.00692*	-0.00713*	-0.0132***	-0.0137***	-0.0223***	-0.0185***	-0.0204***
	(0.00394)	(0.00381)	(0.00397)	(0.00347)	(0.00330)	(0.00276)	(0.00223)	(0.00217)
COVID-19	-0.00834	0.0168	-0.00548	0.00196	0.0383*	0.0302*	0.00974	0.0127
	(0.0239)	(0.0244)	(0.0246)	(0.0231)	(0.0218)	(0.0181)	(0.0113)	(0.0102)
1-month effect	-0.00302	0.0380	0.0149	0.0161	0.0793**	0.0467*	0.0335*	0.0371**
	(0.0379)	(0.0370)	(0.0363)	(0.0341)	(0.0310)	(0.0265)	(0.0189)	(0.0159)
2-month effect	0.00652	0.0556	0.0367	0.0166	0.0938**	0.0442	0.0465**	0.0493**
	(0.0496)	(0.0503)	(0.0489)	(0.0467)	(0.0410)	(0.0317)	(0.0226)	(0.0192)
3-month effect	0.0206	0.0961	0.0985	0.0643	0.143***	0.0910**	0.0880***	0.0819***
	(0.0625)	(0.0628)	(0.0604)	(0.0565)	(0.0477)	(0.0371)	(0.0278)	(0.0224)
4-month effect	0.0581	0.143**	0.145**	0.0996	0.198***	0.137***	0.116***	0.104***
	(0.0716)	(0.0714)	(0.0697)	(0.0685)	(0.0572)	(0.0441)	(0.0325)	(0.0267)
5-month effect	0.0382	0.177**	0.162**	0.113	0.247***	0.195***	0.145***	0.137***
	(0.0860)	(0.0844)	(0.0811)	(0.0798)	(0.0644)	(0.0500)	(0.0373)	(0.0303)
6 plus-month effect	0.0580	0.176*	0.176*	0.153	0.299***	0.219***	0.173***	0.158***
	(0.103)	(0.100)	(0.0946)	(0.0930)	(0.0732)	(0.0570)	(0.0426)	(0.0354)
*Panel B. Regional fixed effects*								
Pre-COVID trend	-0.00585	-0.00698	-0.00842*	-0.0138***	-0.0147***	-0.0224***	-0.0186***	-0.0206***
	(0.00444)	(0.00430)	(0.00437)	(0.00376)	(0.00378)	(0.00306)	(0.00235)	(0.00235)
COVID-19	-0.00515	0.0160	-0.00247	-0.000927	0.0339	0.0330	0.0113	0.0122
	(0.0342)	(0.0337)	(0.0336)	(0.0302)	(0.0301)	(0.0232)	(0.0137)	(0.0116)
1-month effect	-0.00917	0.0279	0.00990	0.00382	0.0649	0.0496	0.0346	0.0363*
	(0.0496)	(0.0488)	(0.0488)	(0.0440)	(0.0438)	(0.0328)	(0.0220)	(0.0188)
2-month effect	-0.00638	0.0410	0.0300	-0.000194	0.0746	0.0515	0.0510*	0.0503**
	(0.0636)	(0.0624)	(0.0625)	(0.0557)	(0.0553)	(0.0398)	(0.0266)	(0.0225)
3-month effect	-0.000241	0.0740	0.0820	0.0377	0.115*	0.0997**	0.0928***	0.0822***
	(0.0765)	(0.0750)	(0.0751)	(0.0658)	(0.0649)	(0.0464)	(0.0312)	(0.0262)
4-month effect	0.0361	0.120	0.127	0.0764	0.174**	0.143***	0.121***	0.103***
	(0.0891)	(0.0874)	(0.0875)	(0.0759)	(0.0749)	(0.0544)	(0.0358)	(0.0297)
5-month effect	0.0363	0.171*	0.161	0.104	0.229***	0.209***	0.156***	0.141***
	(0.102)	(0.0998)	(0.0998)	(0.0863)	(0.0849)	(0.0614)	(0.0405)	(0.0338)
6 plus-month effect	0.0728	0.184	0.186	0.163*	0.298***	0.230***	0.186***	0.161***
	(0.115)	(0.113)	(0.113)	(0.0972)	(0.0956)	(0.0693)	(0.0456)	(0.0384)
Observations	23,116	23,116	23,116	23,116	23,116	23,116	23,116	23,116
Household FE	Yes	Yes	Yes	Yes	Yes	Yes	Yes	Yes
Round FE	Yes	Yes	Yes	Yes	Yes	Yes	Yes	Yes

Author's estimations

Robust standard errors in parentheses. *** *p* < 0.01, ** *p* < 0.05, * *p* < 0.1

## Discussions

In the context of growing interest in the socio-economic impacts of the COVID-19 pandemic, this paper offers insights into short-run impacts of the pandemic on household economic activities and food security outcomes and how households respond to the worldwide aggregate shock in Tajikistan. Emerging literature on the impacts of the COVID-19 pandemic suggests that economic disruptions are inevitable, fueled by the precautionary behavior of households and firms faced with uncertainties even in the absence of lockdowns and containment measures (Brodeur et al., 2020). One of the transmission channels through which the COVID-19 shocks could adversely impact an economy is supply side disruption leading to the halting of production and services, which in turn, lowers the demand for labor. Existing evidence shows increasing unemployment (Adams-Prassl et al. 2020; Béland et al. 2020; Coibion et al. 2020; Forsythe et al. 2020) which results in a drop in household disposable income; this then further decreases consumption and worsens food insecurity during the pandemic.

The findings of this paper are largely consistent with the existing evidence. Five main points are suggested by the results of this paper: (i) household employment rates declined immediately after the first confirmed cases and continued to fall even after 6 months of the pandemic, (ii) consequently, household incomes fell, (iii) food insecurity worsened, (iv) pandemic effects differ depending on location, income and household size, and (v) households increase borrowing and reduce food and health consumption in response to the shock.

Existing studies find that labor market disruptions are mostly driven by lockdowns and social distancing policies (Rojas et al. 2020). Gupta et al. (2020) show that every additional 10 days of a lockdown reduced the employment rate by 1.7 percentage points in the early period of the pandemic in the US. In contrast to these studies, no lockdown has been mandated in Tajikistan, where only voluntary social distancing and precautionary measures were taken. The decrease in the employment rate may be driven primarily by temporary closures of businesses and a reduction in hiring by small establishments. Furthermore, the highly informal nature of small businesses in Tajikistan translates into a lack of job security for those who are employed temporarily by such businesses. My results are consistent with those of Aum et al. (2020) who find that in South Korea, where no lockdowns were mandated, employment declines by 2.7% if infections increase by one per thousand people. The authors claim that employment declines mainly because of reduced hiring by small establishments in food, transportation, and accommodation services. While my results cannot be separated by sectors, closures of businesses in the services and retail sectors in Tajikistan could be linked to a sharp increase in unemployment in the early period of the pandemic.

While the impact of the pandemic on labor market participation has been widely documented, its effects on household income and livelihood have not been investigated as much partly due to a lack of availability of timely data. The majority of the existing studies applied variations of microsimulation to quantify the income and distributional impact of the pandemic. For instance, Diao and Mahrt (2020) show that households that rely on vulnerable non-farm income fell into poverty during a lockdown in Myanmar. Martin et al. (2020) simulate that household savings and consumption drop significantly leading to an increase in poverty from 17.1% to 25.9% in the US after a 3-month lockdown. Compared to these microsimulations or retrospective studies, the advantage of my study is that it uses actual data of both the pre- and post-COVID periods. The fall in the household income appears to be governed by the drops in wages and self-employment incomes, which are likely to be driven by business closures and job insecurities due to the high degree of informality in Tajikistan. Although national and local government-financed organizations are thought to provide the most secure jobs, even these organizations ordered their employees leave without pay for an indefinite period in the early months of the pandemic. All these factors may play a role in reducing household income.

Additionally, this paper adds the broader literature on household coping strategies when faced with income shocks. The literature distinguishes individual or idiosyncratic shocks from aggregate or common shocks, where the former type of shocks affects the individuals or households only while the latter affect the whole community or wider geographical coverage (Dercon, 2002). The idiosyncratic shocks can be insured within the community while the aggregate shocks are cannot. The COVID-19 pandemic is a large worldwide aggregate shock for which many coping mechanisms suggested by the literature including risk sharing, selling assets, and diversifying income sources are not applicable. In the context of the COVID-19, Mahmud and Riley (2021) find that households in Uganda depleted their savings and borrowed more to cope with the COVID-19 shock. Additionally, they find that households decreased their food consumption. My results are consistent with their findings on increased borrowing and reduced food consumption. However, saving depletion is not found to be statistically significant in the Tajikistan’s case, which could indicate the low level of savings among the households in the country.

The results presented in this paper provide evidence that the COVID-19 pandemic adversely impacts household employment, incomes, and food security. Nevertheless, several limitations are worth noting. First, this paper uncovers only the immediate or short-run impacts of the pandemic. Given the ongoing nature of the pandemic, it is difficult to anticipate its long-run impacts and what the shape of the recovery path will be. Studies show that recoveries from past pandemics, such as the 1958 Asian influenza, the 1968 Hong Kong influenza, and the 2002 SARS outbreak, have been V-shaped (Brodeur et al., 2020). However, the economic recovery from the COVID-19 pandemic is not straightforward due to its widespread coverage and the containment policies used to curb the spread of the disease. Second, the impact of the pandemic is likely to be unequal across population groups. Future studies could explicitly explore the differential impacts of the pandemic on various types of households.

## Conclusion

Using simple event studies, this paper quantifies the short-run dynamic causal effects of the COVID-19 pandemic on household employment, income, migration, remittances, and food security. Additionally, it looked at how the effects of the pandemic differ for different types of households and how households cope against the COVID-19 shock.

In general, the results suggest that household employment and income dropped, and food insecurity in Tajikistan worsened in the first months of the pandemic. While all households are affected by the pandemic, the extent of the adverse impacts varies depending on locations, income, and household size. Urban and wealthier households have lost employment and income to a greater extent because they have more employed household members and income prior to the pandemic than rural and lower income households. Although the latter type of households has lost income and employment to a lesser extent, the lost income has greater implications for their food security. In terms of household size, larger households tend to be engaged in self-employment and has suffered income loss more than smaller households that usually are in wage employment.

As the worldwide aggregate shock, the COVID-19 pandemic hit households hard without many coping mechanisms. My results show that households have responded to the COVID-19 pandemic by borrowing more and reducing food and health expenses. The prolonged worldwide pandemic makes it difficult for individuals and households to cope with the shock. With worsening food insecurity, reduced food and health consumption, and income loss, households need social support to survive. The social support is needed not only for the poor households but also for non-poor households as the latter have also suffered significantly in terms of lost employment and income.

Two alternative specifications are tested to check the robustness of the parametric event studies: (i) augmenting the model with covariates, and (ii) including regional fixed effects. The results of the alternative specifications are consistent with the main results.

While the COVID-19 pandemic is ongoing and its full impact is yet to be determined, the findings of this paper provide evidence of the emerging concerns about the impact of the pandemic on household economic activities and food security in developing countries. The results suggest that the implications of the pandemic on households could be devastating even in the absence of government-mandated lockdowns.

## Data Availability

The data that support the findings of this study are available from the World Bank but restrictions apply to the availability of these data, which were used under license for the current study, and so are not publicly available. Data are however available from the authors upon reasonable request and with permission of the World Bank.

## Footnotes

1 The date was chosen to be close to the date of the data used in the paper.

2 https://covid19.who.int (Accessed January 5, 2021).

3 These indicators are selected based on the data availability.

4 There are 25 countries that did not implement any form of lockdowns as of December 2021 (Hale et al 2021).

5 https://asiaplustj.info/en/news/tajikistan/society/20200320/tajikistan-closes-all-its-airports-due-to-coronavirus-concerns (Accessed January 7, 2021.).

6 https://reliefweb.int/report/tajikistan/covid-19-tajikistan-situation-report-4-27-april-2020 (Accessed January 7, 2021.).

7 https://tj.sputniknews.ru/20200511/zhiteli-sogdian-oblast-ostavatsya-dom-koronavirus-1031219299.html (Accessed January 7, 2021.).

8 https://asiaplustj.info/en/news/tajikistan/power/20200521/employees-of-state-institutions-in-dushanbe-began-to-be-sent-on-unpaid-leave (Accessed January 2, 2021.).

9 https://asiaplustj.info/en/news/tajikistan/society/20200320/tajikistan-closes-all-its-airports-due-to-coronavirus-concerns (Accessed January 2, 2021).

10 https://www.themoscowtimes.com/2021/08/12/coronavirus-in-russia-the-latest-news-august-12-a69117 (Accessed January 2, 2021).

11 https://www.rferl.org/a/tajikistan-religion-ramadan-farming-/30573746.html (Accessed January 2, 2021).

12 It is assumed that all households are treated or subject to the COVID-19 disease once their region of residence has confirmed its first community transmission case.
